# *Tritrichomonas Foetus*: A Study of Prevalence in Animal Hosts in Poland

**DOI:** 10.3390/pathogens9030203

**Published:** 2020-03-10

**Authors:** Joanna Dąbrowska, Jacek Karamon, Maciej Kochanowski, Jacek Sroka, Katarzyna Skrzypek, Jolanta Zdybel, Mirosław Różycki, Artur Jabłoński, Tomasz Cencek

**Affiliations:** 1 Department of Parasitology and Invasive Diseases, National Veterinary Research Institute, Partyzantów Avenue 57, 24-100 Puławy, Poland; j.karamon@piwet.pulawy.pl (J.K.); maciej.kochanowski@piwet.pulawy.pl (M.K.); jacek.sroka@piwet.pulawy.pl (J.S.); katarzyna.skrzypek@piwet.pulawy.pl (K.S.); j.zdybel@piwet.pulawy.pl (J.Z.); mrozycki@piwet.pulawy.pl (M.R.); tcencek@piwet.pulawy.pl (T.C.); 2 Department of Large Animal Diseases and Clinic, Warsaw University Of Life Sciences, Nowoursynowska Street 100, 02-797 Warsaw, Poland; artur_jablonski@sggw.pl

**Keywords:** *Tritrichomonas foetus*, cat, cattle pig, wild boar, prevalence

## Abstract

*Tritrichomonas foetus* is described as a pathogen of cattle and cats and also exhibits commensalism with pigs. In order to estimate the prevalence and determine the risk factors for parasite infection, specimens from animal hosts (cat, pigs, and cattle) from Poland were investigated. To our best knowledge, this is the first such study to examine samples from wild boars (*Sus scrofa*) for the presence of *T. foetus.* Data were collected from 117 cats, 172 pigs, 236 wild boars, and 180 cattle. The sensitivity of *T. foetus* identification was increased by using two molecular assays: PCR and loop-mediated isothermal amplification (LAMP). The prevalence of feline tritrichomonosis was 20.51%, and statistically significant differences were obtained between groups of animals regarding age, breed, number of cats, diarrhea, and place of living. Positive PCR and LAMP results for *T. foetus* were estimated for 16.28% of pigs, and the obtained data were significantly correlated with age. Conversely, no significant differences were observed concerning the farm size factor. In our survey, no cases of bovine tritrichomonosis were found, which is consistent with the data from the other countries of the European Union. Similarly, all wild boar samples were also *T. foetus*-negative according to LAMP and PCR.

## 1. Introduction

*Tritrichomonas foetus* (of the order Trichomonadida and family Tritrichomonadidae) is an intriguing protozoan parasite which is pathogenic in cattle and cats and is a commensal organism of pigs.

*T. foetus* is the causative agent of the sexually transmitted disease in cattle called tritrichomonosis, whereupon parasites are placed in the bovine reproductive tract. In cows, trichomonads are found on the epithelial surface of the uterus and vagina, and their presence leads to inflammatory changes, vaginitis, cervicitis, and endometritis [1]. Moreover, *T. foetus* can cause infertility with low pregnancy rates, early embryonic death and, occasionally, abortion. In bulls, trichomonads colonize the preputial cavity without apparent symptoms, becoming a potential source of infection as a carrier and the main reservoir for the parasite [2]. The basic route of transmission is coitus between an asymptomatic male and a susceptible female. Tritrichomonosis in cattle causes serious economic losses, especially in regions with extensive livestock and where natural breeding is used. In Poland, no cases of bovine tritrichomonosis have been found since 1997. However, strict regulations exist to prevent reintroduction of the disease to dairy herds. Moreover, according to the World Organisation for Animal Health (O.I.E.), bovine tritrichomonosis is a notifiable disease on the OIE list of notifiable animal diseases [3,4].

Feline tritrichomonosis was first described about 20 years ago as a gastrointestinal disease with worldwide occurrence [5]. Parasites reside in the large intestine of cats, often along the mucosal surface of the colon, which can lead to lymphocytic and plasmacytic inflammation. Studies have shown that *T. foetus* mainly causes colitis (large bowel diarrhoea), resulting in an increased frequency of defecation, semi-formed to liquid feces and, sometimes, fresh blood or mucus in the feces. Nevertheless, animals suffering from tritrichomonosis retain relatively good body condition, but the disease often manifests as depression, flatulence, abdominal pain, anal irritation, or anorexia [6]. Although cats of all ages can develop parasite infection, this disease is most commonly seen in young cats and kittens, with the majority being under 12 months of age.

Infection may occur in common non-breeding and purebred cats. Additionally, feline tritrichomonosis affects animals from catteries and animal shelters. Infection often develops in animals from catteries and through breeding, where the possibility of parasite transmission by direct contact is very high. Trichomonads are transmitted by the oral–fecal route because they can survive for a few hours in a moist environment [7]. 

*T. foetus* was also identified in the nasal cavity, stomach, and intestines of pigs as a commensal [8]. There is no obligation to examine and report porcine tritrichomonosis, so the real prevalence of parasites in Poland is still unknown. However, according to many authors, *T. foetus* remain present among pig populations [9]. 

There is many speculations on genetic identity of *T. foetus* from animal hosts. Many studies have focused on the identity between the bovine and porcine genotype of *T. foetus*, which was also confirmed by experimental cross-infection. According to Switzer [10], the intravaginal inoculation of trichomonads from pigs resulted in clinical manifestations of the disease in cows. However, dual names of trichomonads in pigs: *Trichomonas suis* or *Tritrichomonas foetus* still exist as synonymous. Following this question, a study conducted by Dabrowska demonstrated the high genetic similarity between the bovine and porcine strains, and showed massive differences in comparison with feline *T. foetus* [11].

According to our best knowledge, an epidemiological survey of *T. foetus* in wild boars (*Sus scrofa*) has not yet been conducted. Despite this fact, wild boars are dangerous reservoirs and vectors of viruses, bacteria, and parasites that can be transmitted to domestic animals and humans [12]. Moreover, the population of wild boars is very abundant in Poland due to the good adaptive capacities of these animals to changing environmental conditions and urbanization. 

The methods used for the diagnostics of tritrichomonosis are mainly based on conventional microscopical methods, which are not useful for all types of samples (i.e., not fresh specimens). Therefore, some molecular methods were adapted for these purposes, among them, conventional PCR [13], real time PCR [14], and also new sensitive methods like loop-mediated isothermal amplification (LAMP) [15]. 

The aim of this study was to determine the prevalence of *T. foetus* infection in selected populations of cats, pigs, wild boars, and cattle in Poland using the molecular tools of PCR and loop-mediated isothermal amplification (LAMP).

## 2. Results

### 2.1. Cats

Among the total 117 feline fecal samples examined in this study, 24 were *T. foetus* positive in LAMP and 21 in conventional PCR. All samples positive in conventional PCR were also positive in LAMP. Additionally, the internal controls showed no inhibition of the PCR reaction in any sample.

The total prevalence, described as the combined results obtained from conventional PCR and LAMP, was 20.51% (95% CI = 13.09–27.94) (Table 1). The percentage of feline tritrichomonosis was estimated for the following infection factors: age, sex, breed, number of cats in one area, diarrhea, and place of living, as shown in Table 2. The highest prevalence of *T. foetus* infection occurred in diarrheic cats (85%), while no positive results were found among non-diarrheic cats. The statistical comparisons between these groups showed significant differences (*χ*^2^ = 9.96, *df* = 1, *p* = 0.001). Moreover, statistically significant differences (*χ*^2^ = 36.49, *df* = 1, *p* < 0.001) were also observed within groups of purebred cats for the prevalence of *T. foetus* (60.7%) and for non-breed cats (7.8%). Cats of the following breeds were positive for *T. foetus* in our study: Maine Coon (5), British (3), Persian (2), Tonkinese (1), Bobtail (1), Sphynx (1), Devon Rex (1), Cornish Rex (1), Siamese (1), and Norwegian Forest (1).

Data on the number of cats revealed a higher percentage of parasites within large (>4 animals) rather than small (≤4 animals) groups of bred animals, with 27.7% and 16.6%, respectively, and these differences were statistically significant (*χ*^2^ = 0.63, *df* = 1, *p* = 0.042). However, a statistically higher prevalence of *T. foetus* infection was observed in younger animals (≤1 year), with 33.3% positivity compared to 14.4% in older animals (>1 year) (*χ*^2^ = 4.23, *df* = 1, *p* = 0.039).

The examined cats were divided into two additional groups: rescue shelter cats and bred cats. The statistical comparisons between the animals showed significant differences in the prevalence of the parasite (*χ*^2^ = 8.83, *df* = 1, *p* = 0.003), with a higher number of positive results.

*T. foetus* results were found in non-shelter cats (31.8%) than in shelter cats with no positive results. Data concerning sex revealed no significant differences between male and female cats.

### 2.2. Pigs and Wild Boars

In our studies, 172 porcine swabs from the nasal cavity were tested. Among these swabs, 28 were positive for *T. foetus* in both assays (Table 1). The total prevalence was 16.28% (95% CI = 10.71–21.85). The internal control was positive in all negative samples. 

We considered 2 other factors, age and farm size, which can have an impact on the prevalence and intensity of *T. foetus* infection (Table 3). The analysis revealed the highest prevalence of parasites (24.3%) in young animals (≤23 weeks) in comparison to adult pigs (>23 weeks). The statistical comparisons between the tested porcine samples showed significant differences (*χ*^2^ = 7.56, *df* = 1, *p* = 0.006). Conversely, no significant differences were observed for farm size. In the case of wild boars, all samples examined by conventional PCR and LAMP gave negative results (Table 1).

### 2.3. Cattle

The preputial fluid from bulls (80 specimens) and swabs from cows (100 specimens) were tested in parallel via cultivation in Diamond medium and using conventional PCR and LAMP.

Microscopic examination of the 4 cultures (2 with swabs and 2 with the fluid) revealed the presence of microorganisms with high morphological similarities to *T. foetus.* However, conventional PCR and LAMP did not confirm the results of the Diamond culture. Moreover, all examined samples from cattle were negative under all molecular methods (Table 1).

## 3. Discussion

To our knowledge, this survey is the first examination of *T. foetus* prevalence in cats, pigs, and wild boars from Poland, and these results indicate that the parasite is present in the feline and porcine population. Worldwide, tritrichomonosis has been reported in cats for several years, with percentages around 2–59% [7]. For instance, studies conducted in Canada revealed that 23.6% of tested cats were *T. foetus*-positive [16]. An examination in the United States estimated the prevalence to be around 38% in feline samples obtained during large-scale hoarding operations [3]. The parasite was also identified in an Asian feline population, with a lower percentage (8.8%) in Japan and Turkey [17,18]. In our study, the percentage of *T. foetus*-positive cats was 20.51%. LAMP was shown to be more sensitive (3 samples more identified as a positive than in conventional PCR). This confirms the higher LAMP effectiveness described earlier by Dabrowska [15].

Moreover prevalence of *T. foetus* among polish cats is similar to the data from other European countries, including France (14.3%) [19], Germany (15.7%) [20], Switzerland (24.4%) [21], and Norway (21%) [22]. The current findings are in agreement with the results obtained in Spain [23], the United Kingdom [24], Greece [25], and China [26] regarding the correlation between the young age of cats (<1 year old) and the occurrence of *T. foetus* infection. However, this correlation is not always observed. For example, in a survey from Italy presented by Holliday [27], 32% of the infected animals with chronic large bowel diarrhoea from a rescue colony were over 18 months old. This is also consistent with the data from Norway obtained by Tysnes [22], where the mean age among the examined healthy cats from cat shows was 29.9 months.

Our results confirm the findings of other authors that the percentage of feline *T. foetus* infection is higher in purebred animals (60.7%) than in non-breed animals (7.8%). There is still a lack of evidence regarding whether some breeds are more predisposed to parasite infection at the genetic or immunological level. Therefore, the higher number of tritrichomonosis cases among purebred cats, which mostly live in multi-cat households, seems to be caused by high-density, and this relationship increases the risk of introducing parasites to catteries, as well as its further transmission, such as by grooming or sharing one litter box. Similarly, our findings indicate a positive correlation between the number of cats being bred and *T. foetus* occurrence. This was also supported by Foster [28] and Miro [23], who confirmed that feline tritrichomonosis is more likely to be present under crowded conditions. Interestingly, we found that the place of living was also an important factor that influenced the presence of the parasite, and in private breeding catteries, the prevalence was higher (24.1%) than in shelters. However, a study conducted by Arranz-Solís [29] showed that there is no significant difference between the number of animals suffering from tritrichomonosis in shelters and breeding centers vs. the animals from one breed. 

Notably, in our study, *T. foetus* infection was detected more frequently within the diarrheic felids (85%), which is not surprising compared to the results obtained in other studies (e.g., Bell [30] and Gookin [31]), where 77% and 70% of positive samples were found, respectively. This finding confirms our conclusions about diarrhea as a primary symptom of *T. foetus* infection in cats. Unfortunately, our questionnaires were incomplete for 51 animals, for which there was a lack of data about the presence and history of diarrhea, especially in the case of rural animals (observations of the frequency of defecation by the owners was likely difficult). 

This survey offers the first evidence of *T. foetus* in Polish pigs. The natural infection of this parasite in pigs was noted previously in other countries, but few reports provide data from examinations of porcine samples. Nevertheless, the presence of *T. foetus* in pig populations can increase the risk of infection for other animal hosts. Therefore, knowledge about the real *T. foetus* prevalence in pigs seems to be very important to prevent the reintroduction of parasites to the cattle population. 

The prevalence found in our study (16.3%) was lower than that in Czech Republic (68–90%) [32], Australia (65%) [33], and Japan (56%) [12]. However, in these other surveys, porcine fecal samples were examined, unlike in our study, in which nasal swabs were tested. The higher prevalence of *T. foetus* in young pigs (≤23 weeks) was consistent with the results revealed by Mueller [34] who identified tritrichomonosis in 10 month old animals. There was no relationship between farm size and the number of infected animals, but we obtained samples from only 10 small farms. Therefore, further studies are warranted in order to survey a greater number of pigs from small farms. 

In our study, *T. foetus* infection was also investigated in wild boars, and there were no positive results among all 236 tested samples. It is difficult to explain this fact, but in our study, the samples were collected from adult animals. This result might be related to the maturity of the immune system producing a higher resistance among adult animals in comparison to young animals. This was partially supported by the results we obtained for pigs, which were consistent with this hypothesis.

In the present epidemiological survey, we estimated the presence of the *T. foetus* parasite in a bovine population. Our results confirm the *T. foetus*-free status of Poland, which is in agreement with the data from the other EU countries [35,36]. The absence of this disease is a consequence of using artificial insemination on a large scale as the main method of cattle reproduction [37]. 

Nevertheless, in the present study, culture examination revealed *T. foetus*-like flagellates in 4 samples (2 swabs and 2 preputial fluid samples). Similar observations were reported by Bernasconi [37] in preputial samples from bulls, suggesting that *Tetratrichomonas* ssp. most likely contaminated the samples. In our study we used two molecular tools for *T. foetus* identification: conventional PCR and LAMP. Both of them are highly sensitive and allowed for detection of DNA equivalent to 1 parasite cell [15]. Moreover, there was no cross reactions with Tritrichomonas-like DNAs (Simplicimonas sp., *P.hominis*) [15]. Therefore, both molecular tests have not confirmed the presence of *T. foetus* among Polish bovine samples.

In conclusion, our survey demonstrates the presence of *T. foetus* in cat and pig populations from Poland. Moreover, the strong association between parasite infection and risk factors like age, breed, number of cats, diarrhea, and place of living was estimated. Similarly, a higher prevalence of *T. foetus* was obtained in the young porcine group. There were no *T. foetus*-positive results among the wild boar and cattle samples included in our studies. Therefore, despite Poland being considered free of bovine tritrichomonosis, and taking into account the genetic similarity, the occurrence of *T. foetus* in pigs may increase the risk of *T. foetus* transmission to cattle.

## 4. Material and Methods

### 4.1. Study Design and Specimens from Animals

#### 4.1.1. Cats

A total of 117 freshly voided feline fecal samples from the area of Poland were delivered between September 2015 and July 2017 by owners who wanted to participate in the studies. Samples were obtained from 9 provinces: Dolnośląskie, Kujawsko-pomorskie, Lubelskie, Małopolskie, Mazowieckie, Pomorskie, Świętokrzyskie, Warmińsko-mazurskie, Wielkopolskie. Further information about the cats was included in the questionnaires. Only feline samples in which complete data had been provided by the owners were included in our analysis. Based on this data, the cats were divided into groups based on various factors: age, sex, breed, number of cats, diarrhea, place of living. All samples were frozen and stored at −20 °C until DNA extraction.

#### 4.1.2. Pigs and Wild Boars

The study included 172 (rinsed into 0.9% NaCl) swabs from the nasal cavities of pigs, which were collected between 2015 and 2017. The samples were provided from slaughterhouses and veterinary clinics from provinces: Kujawsko-pomorskie, Pomorskie, and Zachodniopomorskie. Moreover, the data concerning age and farm size were provided by the owners. According to these data, the pigs were sorted into 4 groups based on age and farm size. All samples were frozen and stored at −20 °C until DNA extraction.

A total of 236 samples from wild boars were obtained for our studies. Swabs (rinsed into 0.9% NaCl) from nasal cavities were taken from animals hunted in 11 provinces (Dolnośląskie, Kujawsko-pomorskie, Lubelskie, Łódzkie, Mazowieckie, Opolskie, Podkarpackie, Pomorskie, Śląskie, Warmińsko-mazurskie, and Zachodniopomorskie) between 2013 and 2014. All samples were frozen and stored at −20 °C until DNA extraction.

#### 4.1.3. Cattle

A total of 180 bovine specimens (rinsed into 0.9% NaCl) were obtained for our studies during routine *T. foetus* examinations. Preputial smegma from bulls and swabs from cows were submitted to the Department of Parasitology, NVRI, in Pulawy between 2015 and 2017. Bovine samples were obtained from 7 provinces: Lubelskie, Lubuskie, Łódzkie, Mazowieckie, Podlaskie, Pomorskie, Świętokrzyskie. All bovine samples were examined microscopically and inoculated into Diamond media according to the method described in the OIE manual [4]. The remaining volume of specimens was frozen and stored at −20 °C until DNA extraction.

### 4.2. Examination of Samples

#### 4.2.1. DNA Extraction

Samples from cattle (swabs and preputial fluid with 0.9% NaCl), pigs, and wild boars (swabs from nasal cavity with 0.9% NaCl) were centrifuged at 400× *g* for 4 min and 100 µL of sediment were used for isolation of *T. foetus* genomic DNA. Specimens were extracted following the protocol of the DNeasy^®^ Blood and Tissue Kit (Qiagen, Germany) for cultured cells. In the last step DNA of *T. foetus* was eluted in 200 μL elution buffer. DNA isolation from samples of wild boars were performed with the Genomic Mini (A&A Biotechnology, Gdynia, Poland) using the protocol for cultured cells. DNA was eluted in 200 μL elution buffer. Genomic DNA from fecal feline samples were extracted according to the protocol with using the ZR Fecal DNA MicroPrep^®^ (Zymo Research, Irvine, CA, USA) kit. DNA was eluted in 100 μL elution buffer. All DNA samples were stored at −20 °C. 

#### 4.2.2. DNA Amplification

The sensitivity of *T. foetus* identification increased by using two molecular assays: conventional PCR and LAMP. All extracted *T. foetus* DNA samples were subjected to PCR according to Felleisein [12], with an amplification product size of 347 bp. The sequence amplified by PCR was a part of the ribosomal 5.8S rRNA, ITS1, and ITS2. The PCR amplification was performed in a 50 µL reaction mixture including 1 pmol of each primer (TRF3 and TRF4), 200 µL of each dNTP (Fermentas, Germany), 250 U Taq polymerase (Qiagen, Germany), 5 µL 10× concentrated PCR buffer (Qiagen, Germany), 28.6 µL of DNAse-free water (Fermantas, Germany), and 1 µL DNA. The PCR was performed with 1 cycle of 94 °C for 30 s, 40 cycles of 67 °C for 30 s, 72 °C for 90 s, and a final extension step of 72 °C for 15 min. For the detection, 5 μL of PCR product was electrophoresed on 2% agarose gels using a Mini-SubCell GT chamber and Power Pac Basic (Bio-Rad). After electrophoresis, the gel was bathed in a 0.5% ethidium bromide solution, and the results were visualized using GelDoc (Bio-Rad) with the Quantity One (Bio-Rad) software. 

The TF-βtub-LAMP [14] was used as the second molecular method for *T. foetus* identification. The reaction was performed in 15 µL total volume and included 7.5 μL of Isothermal Mastermix (OptiGene, Horsham, UK), 1 μL of each primer (20 pmol TF-βtub-FIB/TF-βtub-BIP and 5 pmol TF-βtub-F3/TF-βtub-B3), 1.5 μL of PCR-grade H_2_O (Qiagen, Germany), and 2 μL of the DNA template. Amplification was carried out in a Biometra thermocycler (Gottingen, Germany) at 65 °C for 1 h. After amplification, 1 μL 1:100 SYBR Green I (Invitrogen, Carlsbad, CA, USA) was added for each LAMP product. The results were assessed based on color changes (a positive result was considered green and negative was red). Furthermore, 1 μL positive control DNA (isolated from the *T. foetus* reference strain ATCC 30924) was added to duplicates of all tested samples in order to monitor the possible inhibitory effects of both methods (i.e., an internal control).

#### 4.2.3. Statistical Analysis

The statistical analysis was performed using Statistica v10 (StatSoft Inc., Tulsa, OK, USA). The prevalence was calculated as the percentage of animals (cats and pigs) in which *T. foetus* was identified. Moreover, 95% confidence intervals (95% CI) of the prevalence of infection intensity were calculated. Differences in the prevalence of the factors between groups of cats were estimated by a chi-square test and were considered to be statistically significant at *p* < 0.05.

## Figures and Tables

**Table 1:** Prevalence of *T. foetus* in animals hosts.

Animals	Numbers of Tested Animals	Numbers of *T. foetus* Positive Animals Conventional PCR	Numbers of *T. foetus* Positive Animals LAMP	Total Prevalence [%] (95% CI)
cats	117	21	24	20.51 (13.09–27.94)
pigs	172	28	28	16.28 (10.71–21.85)
wild boars	236	0	0	0.0
cattle	180	0	0	0.0

**Table 2:** Influence of selected factors on the presence of *T. foetus* in cats.

Factors	Numbers of Examinated Cats	Groups	Numbers of Cats in Groups	Numbers of *T. foetus* Positive Cats (LAMP)	[%] (95% CI)	*p*-Value
Age	112	≤1 year	36	12	33.3 (17.1–49.5)	0.039
		>1 year	76	11	14.4 (6.3–22.5)	
Sex	117	male	55	13	23.6 (12.0–35.2)	0.430
		female	62	11	17.7 (7.9–27.5)	
Breed	117	purebred	28	17	60.7 (41.4–80.0)	0.000
		non-breed	89	7	7.8 (2.1–13.5)	
Number of cats	66	≤4 cats	12	2	16.6 (0.0–41.4)	0.042
		>4 cats	54	15	27.7 (15.4–40.1)	
Diarrhoea	66	presence of diarrhoea	20	17	85 (67.8–100)	0.001
		without diarrhoea	46	0	0.0	
Place of living	117	breeding	87	21	24.1 (14.9–33.3)	0.003
		rescue shelter	30	0	0.0	

%—prevalence; 95% CI—95% confidence interval; *p*—compares cat groups within factors with Chi-Square.

**Table 3:** Influence of selected factors on the presence of *T. foetus* in pigs.

Factors	Numbers of Examinated Pigs	Groups	Numbers of Pigs in Groups	Numbers of *T. foetus* Positive Pigs	[%] (95% CI)	*p*-Value
**Age**	**172**	young (≤23 weeks)	82	20	24.3 (14.8–33.8)	0.006
		adult (>23 weeks)	90	8	8.8 (2.8–14.8)	
**Farm size**	**172**	small	10	2	20 (0–5)	0.740
		large	162	26	16 (10.3–21.7)	
